# Co-occurrence of IgA nephropathy and IgG4-Tubulointersitial nephritis effectively treated with tacrolimus: a case report

**DOI:** 10.1186/s12882-021-02477-w

**Published:** 2021-08-12

**Authors:** Mi Tian, Junjun Luan, Congcong Jiao, Qing Chang, Jeffrey B. Kopp, Hua Zhou

**Affiliations:** 1 Department of Nephrology, Shengjing Hospital of China Medical University, 36 Sanhao St, Shenyang, 110004 Liaoning China; 2 Clinical Epidemiology, Shengjing Hospital of China Medical University, Shenyang, China; 3 Renal Diagnostics and Therapeutics Unit, NIDDK/NIH, Bethesda, MD USA

**Keywords:** IgA nephropathy, IgG4-related tubulointerstitial nephritis, Tacrolimus, Serum IgG4

## Abstract

**Background:**

Cases of concurrent immunoglobulin A nephropathy (IgAN) and IgG4-related tubulointerstitial nephritis (IgG4-TIN) are rare and previous case reports have lacked important data. KDIGO suggests a treatment with systemic glucocorticoids in IgAN patients. Glucocorticoids are recommended as the first-line therapy for IgG4-TIN. The use of tacrolimus as a long-term maintenance treatment has not been described. We report the case of a man who developed IgAN and IgG4-TIN without abnormalities in extra-renal tissue, without renal function abnormalities or impairment as well, and was treated by tacrolimus as a long-term maintenance during 45 months follow-up.

**Case presentation:**

A 56-year-old Chinese man first presented to our hospital with the chief complaint of foamy urine for 1 year and hematuria for 3 months, with a medical history of hypertension. Testing revealed a notable increase in serum IgG4 level without abnormalities in renal function or imaging, or in dysfunction other organs. Renal biopsy showed mesangial extracellular matrix proliferation, increased mesangial cell numbers and infiltration of plasma cells. Immunofluorescence showed mesangial positivity for IgA and C3. Immunohistochemistry staining showed widespread IgG4 and increased CD38 and CD138 expression. Electron microscopy showed immune complexes located on the tubular basement membrane. He was diagnosed with IgAN and IgG4-TIN. He received glucocorticoids, leflunomide and tacrolimus to induce remission. He was given tacrolimus as long-term maintenance treatment. When tacrolimus was temporarily withdrawn, proteinuria recurred. After resuming tacrolimus therapy, he again entered complete remission. After 45 months of therapy, he remains in complete remission and the serum IgG4 level is normal.

**Conclusions:**

The finding of concurrent IgAN and IgG4-TIN without abnormalities in renal function, imaging or extra-renal tissue is rare and their coexistence may be coincidental. Long-term treatment with tacrolimus proved effective and he has remained in remission during 45 months follow-up.

## Background

IgA nephropathy (IgAN) is the most common cause of primary glomerulonephritis worldwide [1], and is particularly common among Asians [2]. Predominant IgA deposition in the glomerular mesangium by biopsy has been used as the defining characteristics for the diagnosis of IgAN [3]. IgAN was the most common glomerulopathy, with a frequency of 28.1% [4].

IgG4-related disease (IgG4-RD) is an fibroinflammatory condition involved multiple organs characterized by IgG4 positive plasma cells infiltration in the involved tissues and elevated serum IgG4 level [5], with a prevalence of IgG4-RD in Japan estimated as 0.28–1.08/100,000 people in 2012 [6]. IgG4-related tubulointerstitial nephritis (IgG4-TIN), is the common manifestation of IgG4-related kidney disease (IgG4-RKD), accounting for about 15–25% of all IgG4-RD [5, 7].

Glomerular disease in patients with IgG4-RD has been reported in the setting of IgG4-TIN, but most such patients had extrarenal involvement and multiorgan involvement [8–10]. Only one case co-existing IgAN and IgG4-TIN has been reported, with dacryoadenitis and sialadenitis, but treatment was not discussed [5]. The co-occurrence of IgAN and IgG4-TIN without extrarenal involvement has not been previously reported. While glucocorticoids are recommended as the first-line therapy for IgG4-TIN, the role of tacrolimus as a long-term maintenance treatment has no report. Tacrolimus effectively reduces proteinuria in IgAN [11]. Here we report the case of a man who developed IgAN and IgG4-TIN without extra-renal manifestations and was treated with tacrolimus as maintenance therapy during 45 months of follow-up.

## Case presentation

### Clinical history and initial laboratory data

A 56-year-old Chinese man was admitted with the chief complaint of foamy urine for 1 year and hematuria for 3 months. Medical history was notable for hypertension for 5 years; the highest blood pressure was 180/110 mmHg. His blood pressure was poorly controlled, 140–150/90–100 mmHg, while he took candesartan irregularly.

He denied diabetes, hepatitis, tuberculosis, and coronary heart diseases. His medical family history was unremarkable. He denied use of illicit drugs and exposure to pesticides and other toxins. Medications included an angiotensin-converting enzyme inhibitor. His weight was 79 kg, blood pressure was 170/90 mmHg, and physical examination was otherwise unremarkable.

**Fig. 1:**
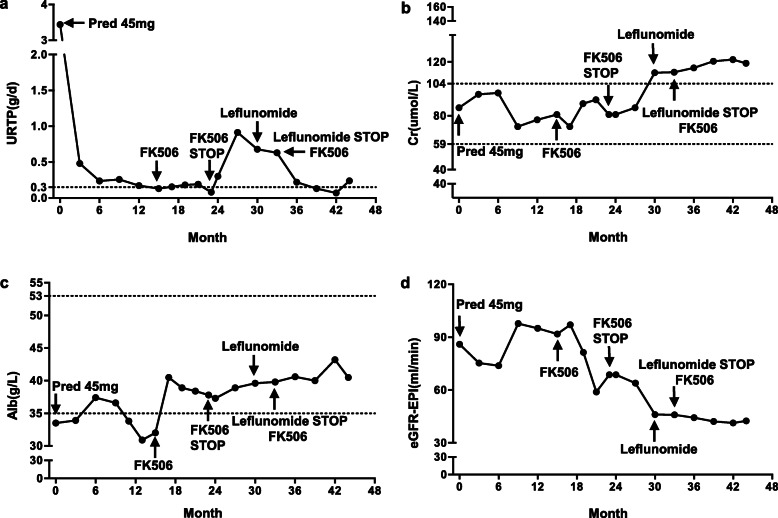
Clinical course of kidney disease activity after admission. Urinary total proteinuria (URTP) (**a**); creatinine (Cr) (**b**); serum albumin (Alb) (**c**); eGFR-EPI (**d**) based on the administration or stopping of glucosteroid and immunosuppressants

On admission, laboratory data showed urinary total proteinuria (URTP) 3.4 g/d, serum total protein 75.3 g/l, serum albumin (Alb)33.5 g/l, serum creatinine (Cr) 86 umol/l, (suggesting an eGFR of 87 ml/min/1.73m^2^ by the CKD-EPI equation) (Fig. 1), and C-response protein (CRP) increased at 21.90 mg/l. Urinalysis showed hematuria with 482 red blood cells (RBC) per high-power field, with 80% dysmorphic RBC.

**Fig. 2:**
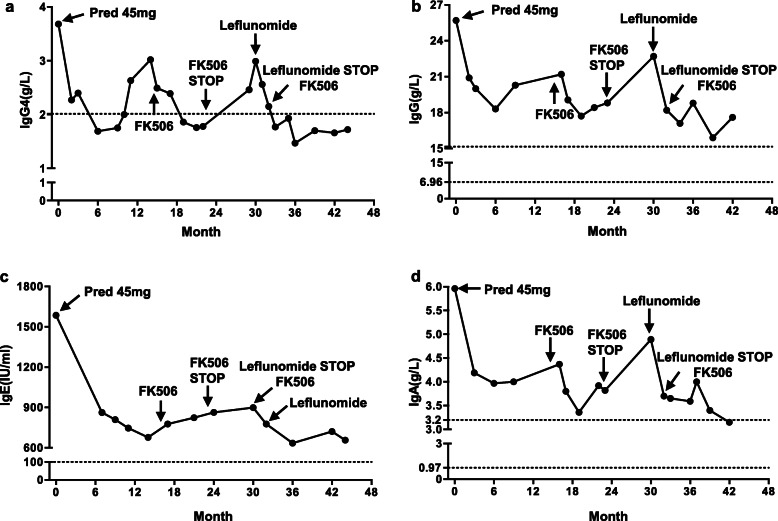
The serum levels of serum IgG4-RD related immunoglobulins after therapy. IgG4 (**a**); IgG (**b**); IgE (**c**). serum IgA (**d**). Both tacrolimus and leflunomide reduced serum IgG4 levels

Clinical immunology tests revealed the following: anti-nuclear antibody (+), anti-neutrophil cytoplasmic antibodies (−), IgG4 3.68 g/l, IgG 25.70 g/l, IgA 5.96 g/l, IgM 1.41 g/l, IgE 1586 IU/ml (Fig. 2), complement 3 (C3) 0.99 g/l, C4 0.20 g/l, CRP 46 mg/l, and erythrocyte sedimentation rate 58 mm/h. Serum immune electrophoresis, glucose, thyroid function, and tumor markers were all normal.

**Fig. 3:**
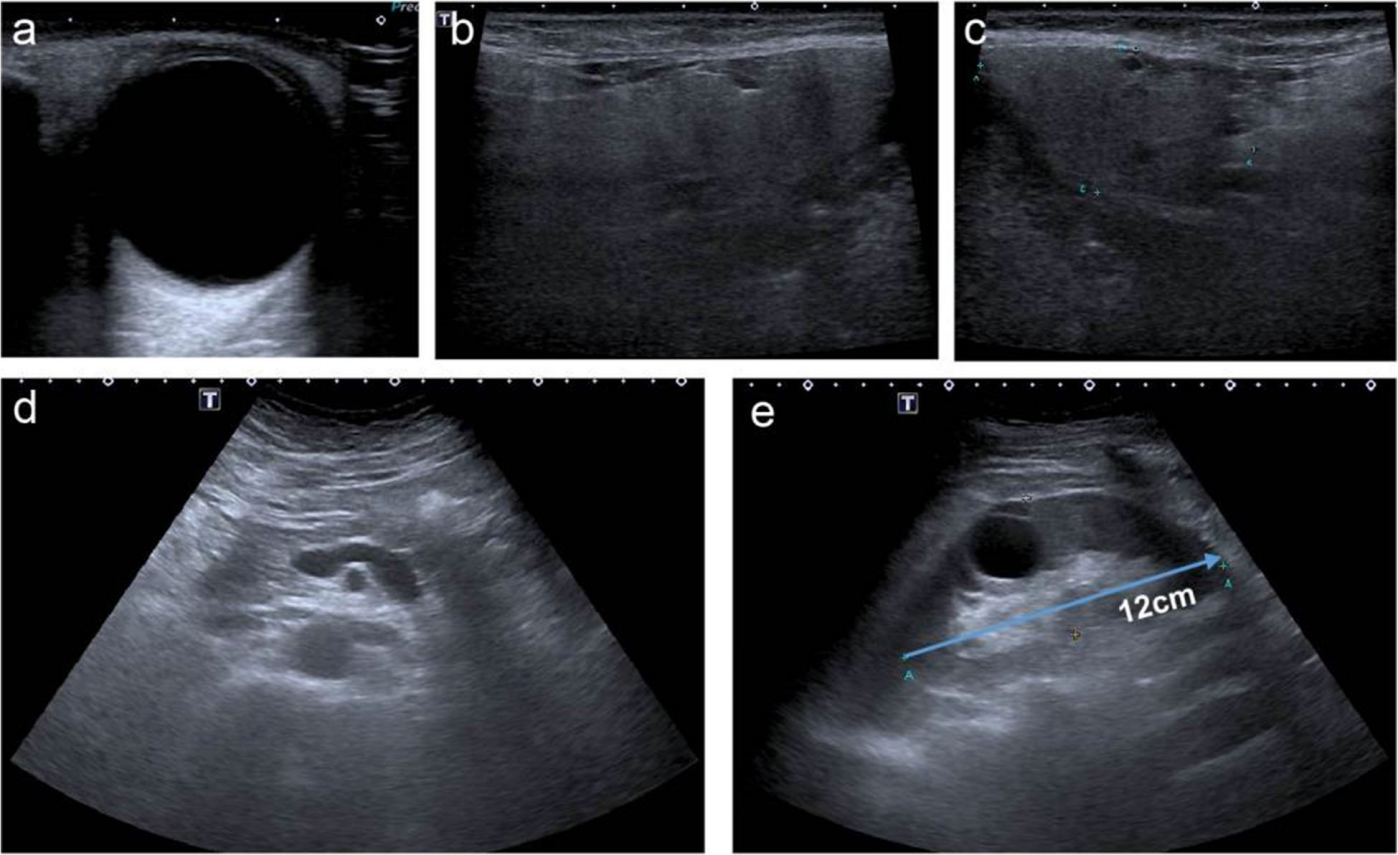
Ultrasound images of extrarenal and renal manifestations. No enlargement of lacrimal gland (**a**), parotid gland (**b**), submandibular glands (**c**), pancreas and retroperitoneal fibrosis (**d**). Normal size kidney and renal cortex thickness and ureteral inflammatory pseudotumor (**e**) at initial admission

Evaluation for infectious disease was negative, including serologies for hepatitis, HIV, and syphilis. Chest computerized tomogram (CT) scan and enhanced abdominal CT scan were normal. Renal ultrasound showed left kidney dimensions was 12.61 cm in height 5.58 cm in width and 5.43 cm in depth, while the right kidney dimensions were 11.22 by 5.62 by 7.86 cm. Both kidneys were slightly enlarged and hyperechoic (Fig. 3).

### Kidney biopsy

**Fig. 4:**
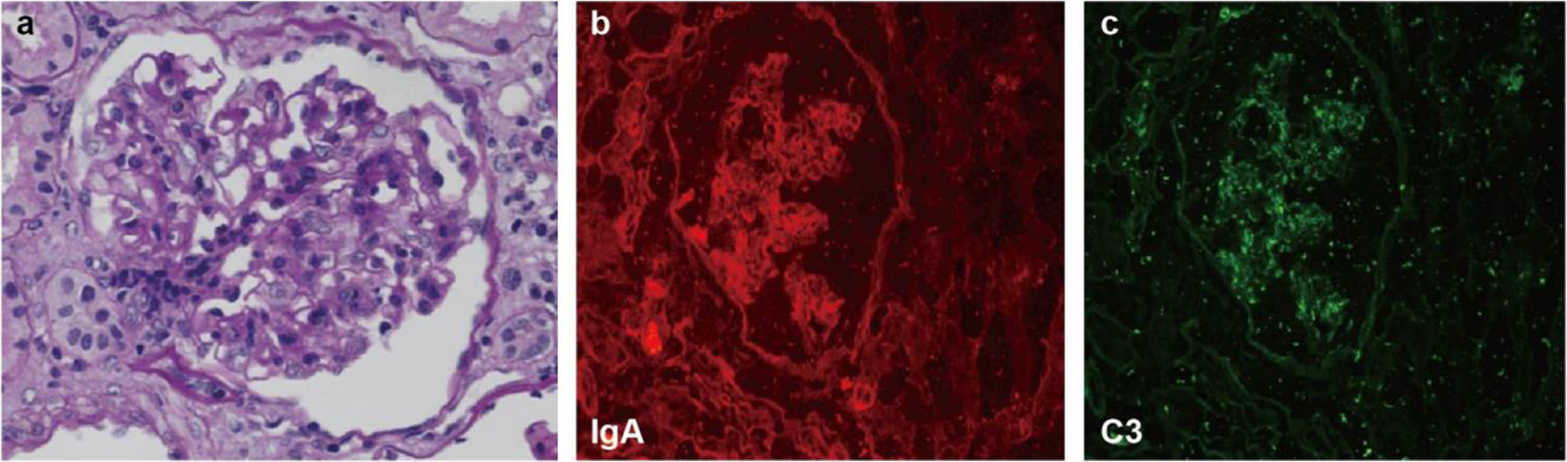
Typical pathological features of IgA nephropathy in renal biopsy. Total 14 glomeruli were present in the renal biopsy specimen. Glomeruli exhibited proliferation of mesangial cells and increased mesangial matrix with no apparent intraductal hyperplasia or crescentic lesions on periodic acid-Schiff (PAS) staining (**a**). Immunofluorescence staining demonstrated strongly positive punctate staining within the mesangium for IgA (**b**) and C3 (**c**). Original magnification 400 ×

On periodic acid Schiff (PAS) staining under light microscopy, one out of fourteen glomeruli showed global sclerosis. The mild to moderate mesangial proliferation was present. The two glomeruli showed ischemic shrinkage (Fig. 4a). There was no endocapillary hypercellularity, fibrosis of Bowman’s capsule wall, or glomerular crescents. A few glomeruli manifest adhesion of the capillary tuft to Bowman capsule.

**Fig. 5:**
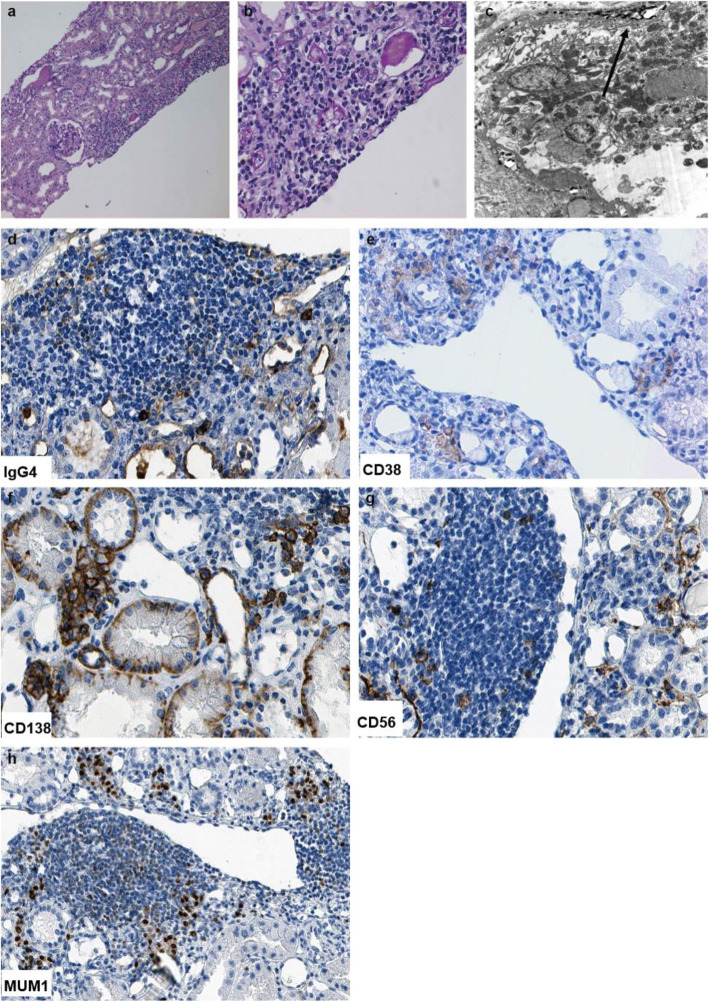
Typical pathological features of IgG4-Tubulointersitial nephritis and immunohistochemistry staining of surface biomarkers of plasma cells in renal biopsy. The renal interstitium is infiltrated by plasma cells and lymphocytes predominantly with fibrosis on periodic acid-Schiff (PAS) staining. Original magnification 100x (**a**) and 400× (**b**). TBM electron-dense deposits were seen under electron microscope. Original magnification 8000x (**c**). Marked increase in IgG4-positive plasma cells was seen in the infiltrated cells on immunohistochemistry staining. Original magnification 400× (**d**). CD38-positive plasma cells (**e**), CD138-positive plasma cells (**f**), CD56-positive plasma cells (**g**), and MUM1-positive plasma cells (**h**) were seen in the interstitium on immunohistochemistry staining. Original magnification 400 ×

Renal tubules exhibited multifocal atrophy and proteinaceous casts. Renal tubular epithelial cells showed granular and vacuolar degeneration. Focal interstitial fibrosis and interstitial edema with significant inflammatory cell infiltration, including lymphocytes and plasma cells were present, (Fig. 5a, b). Occasional hyaline degeneration in arteriolar walls was observed.

On immunofluorescence staining, a pattern of lumpy-like deposition was seen in the mesangium (for the following: IgA was 3+, C3 was 3+, while immunostaining for C1q, fibrinogen, IgG, IgM was negative (Fig. 4b and c).

The elevated serum IgG4 level led us to further examine the infiltrated monocytes in renal biopsy. The absolute number of positive IgG4+ cells per high power field> 10 (Fig. 5d). As surface biomarkers of plasma cells, positive staining of CD38, CD138, CD56, and MUM1 were seen in the interstitium (Fig. 5e-h).

Electron micrographs also revealed immune complexes deposited on tubular basement membranes (Fig. 5c).

### Diagnosis of IgAN and IgG4-TIN

This patient manifested typical features of IgAN, including hematuria, proteinuria, mesangial cell proliferation, mesangial matrix expansion, and positive immunofluorescence staining of mesangial IgA and C3 deposits [3]. Plasma cells were identified on PAS-stained sections and EM demonstrated immune complexes on basement membranes. CD38+/CD138+ plasma cells in kidney expressed IgG4. The absolute number of IgG4+ cells per high power field was > 10. Serum IgG4 level was elevated, with values of 3.7 g/l. The size of kidney was enlarged.

In summary, the combination of clinical, serologic, radiologic, and pathologic data, in this patient fulfilled the 2019 American College of Rheumatology and European League Against Rheumatism (ACR/EULAR) criteria for the diagnosis of IgG4-renal disease [12].

### Treatment course and clinical follow up

After the diagnosis of IgAN and IgG4-TIN was made, prednisolone (45 mg/d) therapy was initiated, based on the KDIGO guideline for IgAN [13] therapies included the Chinese herb *Tripterygium wilfordii*, angiotensin converting enzyme-inhibitor was replaced by an angiotensin receptor blocker, together with amlodipine to maintain his blood pressure below 130/80 mmHg. After 12 weeks, the prednisolone reduced gradually and proteinuria level reached complete remission (< 0.3 g/d), and *Tripterygium wilfordii* was administered for 18 months maintenance (Fig. 1).

From the 15th month on, serum IgG4 and IgA re-elevated as well as serum albumin dropped. Tacrolimus was put on the patient as immunosuppresent based on a Cochane systematic review [14]. Before tacrolimus was chosen, other traditional immunosuppressants were also considered. The patient declined intravenous cyclophosphamide due to the inconvenience of hospitalization and concern for tumor occurrence. Mycophenolate mofetil was excluded because the increased local risk for *Pneumocystis carinii* infection which requires sulfamethoxazole, which can also cause interstitial nephrititis. Rituximab therapy was not available for this patient because of his financial reasons.

After 3 months of tacrolimus treatment, kidney disease activity and immune indices were remitted again for 8 months (Fig. 1). When the COVID-19 pandemic emerged and the patient was no longer able to travel to our hospital. His local physician stopped tacrolimus from the 30th month and replaced it with leflunomide for 2 months. Both renal diseases relapsed. When he was able to return to our clinic at 32th month, tacrolimus was administered again. After 3 months treatment with tacrolimus, he again entered complete remission and the remission remains over 45 months of follow up (Figs. 1 and 2) as of this writing.

**Fig. 6:**
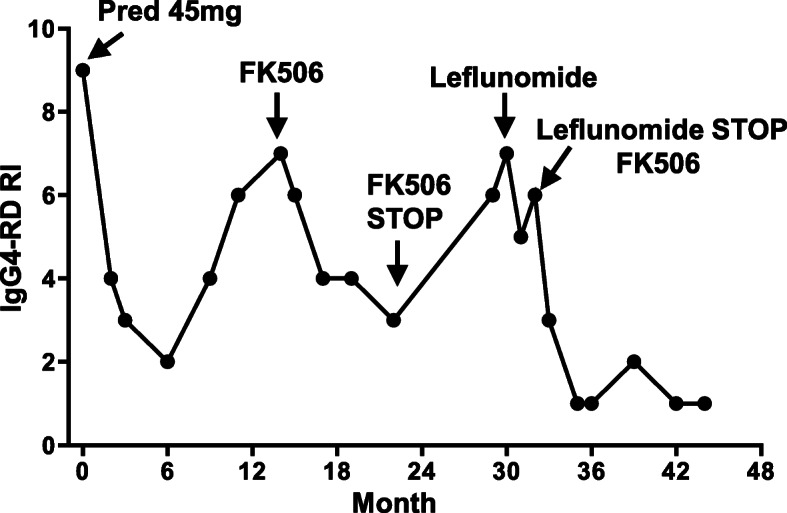
The dynamic changes of IgG4-RD Responder Index (RI) from the intitially presentation to 45 mouths of follow-up. Tacrolimus reduced the scores of IgG4-RD RI

At the most recent visit in June 2021, URTP remained < 0.3 g/d, IgG4 plasma was negative (Figs. 1 and 2). In addition, the IgG4-RD Responder Index (RI) was calculated and revealed the suppression of IgG4 production (Fig. 6). Although abnormalities in renal function was present with normal size and cortical thickness of the kidney, but no extrarenal lesions appeared, such as gland swelling, lymphadenopathy and retroperitoneal fibrosis compared to those images at the initial presentation of the kidney disease was diagnosed.

## Discussion and conclusions

This study reported a man patient with concurrence of IgAN and IgG4-TIN without renal function abnormalities or impairment at the initial hospitalization. At admission, the patient presented marked proteinuria, the decreased serum albumin level, and normal renal function. The renal biopsy showed typical mild-moderate mesangial proliferation, predominant IgA, and C3 deposition. However, abundant monocytes infiltrated in the tubule-interstitium of the kidney biopsy and serum IgG4 level increased near two-fold. On immunohistochemistry of renal biopsy, absolute number of positive IgG4 cells was more than 10/high power field and plasma surface biomarkers were positive. Concurrence of IgAN and IgG4-TIN was diagnosed. Oral prednisone and tacrolimus showed effective for both IgAN and IgG4-TIN with over than 45 months follow up (Figs. 1 and 2).

Takako Saeki et al. firstly reported a patient biopsy proven as IgG4-TIN without prominent proteinuria and microscopic hematuria. However dominant mesangial IgA deposition is also seen in one case [5]. This case is IgG4-TIN predominant with an additional IgA deposition. The abnormality is mild in urine analysis. Our patient presented typical clinical nephritis syndrome with predominant proteinuria, microscopic hematuria, and decreased serum albumin level. Renal biopsy revealed a typical IgAN. However, large amount of the infiltrated lymphocytes led us to think about a possible co-existing tubule-interstitial disease. With blood examination, serum IgG4 level was elevated. Further immunohistochemistry staining showed plenty positive IgG4 cells and positive cells stained with multiple plasma surface biomarkers such as CD38, CD138, CD56, and MUM1 (Fig. 5). Even though both case presented similar co-concurrence of IgAN and IgG4-TIN. The clinical kidney disease activity was quite different. Saeki et al. reported the rare pathological co-existing phenomenon of both diseases. We followed up over 45 months to explore the effective treatment when kidney disease showed significant active.

IgAN is the most common glomerulonephritis in the world [4] and it is easy to be diagnosed if biopsy is available. IgAN is diagnosed by the presence of mesangial dominant IgA staining on immunofluorescence and mesangial hyper-cellularity by light microscopy [15]. Our patient presented as a feature of a typical IgAN. On the other hand, IgG4-RD is a multi-organ immune-mediated condition associated with fibroinflammatory lesions [16, 17]. In 2019, ACR/ EULAR made new diagnostic criteria for IgG4-RD, providing a more specific and sensitive scoring system [12]. IgG4-related kidney disease (IgG4-RKD) is a comprehensive term for the renal lesions associated with IgG4-RD [18]. IgG4-related tubulointerstitial nephritis (IgG4-TIN) is the most common renal manifestation of IgG4-RKD [19, 20]. The diagnosis of IgG4-TIN is based on the presence of four criteria: (1) plasma cell-rich TIN, with renal IgG4-positive plasma cells > 10 / high power field; (2) an increased IgG4+/IgG+ plasma cell ratio (> 40%) in the most concentrated field; (3) TBM immune complex deposits in the tubular basement membrane, identified by immunohistochemistry, immunofluorescence, and/or electron microscopy; and (4) at least one imaging feature (multiple cortical low-density nodules, round wedge-shaped lesions on enhanced CT, or diffuse kidney enlargement), OR characteristic serologic abnormality (most commonly elevated total IgG or IgG4 level), OR other organ involvement [6, 8]. Our case met the above criteria and was diagnosed as IgG4-TIN. We firstly reported this case with co-concurrence of dominant IgAN and IgG4-TIN without extra-renal organ impairment.

In terms of the treatment, KDIGO suggested a treatment course of systemic glucocorticoids in IgAN patients with proteinuria above 1 g/day and eGFR higher than 50 ml/min/1.73 m^2^ despite supportive care. The benefit of immunosuppressive agents remains controversy [21]. No randomized clinical trials have been evaluated and compared the efficacy of different treatment regimens for IgG4-RD [19]. Glucocorticoids are recommended as the first-line therapy for IgG4-RKD [19, 22]. However, to avoid steroid resistance or relapse at discontinuation and long-term undesirable side effects, other steroid sparing agents, such as B cell depleting agents with example of rituximab, azathioprine, MMF and cyclophosphamide [23], are reasonable choices for second-line agents. *Tripterygium wilfordii*, a traditional Chinese medicine, was beneficial for numerous Chinese IgAN patients [24]. Considering the side effects of long-term steroid, we used *Tripterygium wilfordii* as the treatment for IgAN in first 18 months. When we followed up this patient on 15th month, tacrolimus was administered due to the elevated serum IgG4, IgA and total IgG levels, and the decreased albumin level indicating the activity of the disease. Recent meta-analysis and RCTs showed that tacrolimus was beneficial for the remission of proteinuria in patients with IgAN, showing that tacrolimus may be a promising agent for IgAN [11, 25–29]. IgAN mainly contributed to prominent proteinuria for this patient. Our patient showed a good response to tacrolimus indicating as proteinuria reduction and serum albumin recovery (Fig. 1). With the diagnosis of IgAN, co-existing IgG4-TIN was diagnosed. A recent retrospective study reported the effectiveness of tacrolimus in five relapsed IgG4-RD patients without increasing glucocorticoids [30]. T follicular helper cell is involved in the pathogenesis of IgG4-RD [31–33]. Tacrolimus can prevent calcineurin activation and block dephosphorylation of nuclear factor of activated T cells, and specifically suppresses both lymph nodes and circulating T follicular helper cells. Thus, tacrolimus can be a reasonable choice for IgG4-RD. Tacrolimus treatment can be used for either reducing IgAN proteinuria [11, 25–29] or serum IgG4 level [30–33]. These data provided us evidence to use tacrolimus to treat co-existing IgAN and IgG4-TIN condition. Our patient showed good response to tacrolimus with a comprehensive evaluation using the reduction of IgG4-RD RI values (Fig. 6), which was demonstrated to be a practical, reliable, and responsive tool for assessing the progression of IgG4-RD [34–36]. During the treatment, the local physician stopped tacrolimus and put leflunomide on him, when the patient reach completed remission. Leflunomide was reported to reduce kidney damage of IgAN patients [37] and to be effective in tapering glucocorticoids and maintaining glucocorticoid-induced remission in IgG4-RD [38], However, our patient showed failure of maintaining remission by leflunomide. We reused tacrolimus to replace leflunomide. The patient again entered complete remission 3 months after resuming tacrolimus. Fortunately, the patient remained the remission over 45 months follow-up. It is important to watch out the possible IgAN and IgG4-TIN and to take a continuous and reliable close follow-up when tacrolimus is used for a treatment regimen for this condition.

In summary, we presented a case of a 56-year-old male without renal function abnormalities or impairment who was diagnosed as concurrence of IgAN and IgG4-TIN without extrarenal involvement and follow up 45 months. Tacrolimus was effective for the both diseases. In the early stage of IgG4-TIN without extrarenal involvement, tacrolimus was beneficial for development of the extrarenal tissue impairment by controlling serum IgG4 level and was also effective for IgAN.

## Data Availability

The datasets used and/or analyzed during the current study are available from the corresponding author on reasonable request.

## Abbreviations

ACR: American College of Rheumatology
Alb: Albumin
C3: Third complement component
Cr: Creatinine
CR: Complete remission
CRP: C-response protein
EULAR: European League Against Rheumatism
IgAN: Immunoglobulin A nephropathy
IgG4-RD: IgG4-related disease
IgG4-RKD: IgG4-related kidney disease
IgG4-TIN: IgG4-related tubulointerstitial nephritis
MN: Membranous nephropathy
PAS: Periodic acid Schiff
RBC: Red blood cells
RI: Responder index
URTP: Urinary total proteinuria

## References

[1] Schena F, Nistor I (2018). Epidemiology of IgA nephropathy: a global perspective. Semin Nephrol.

[2] Kiryluk K, Li Y, Scolari F, Sanna-Cherchi S, Choi M, Verbitsky M, Fasel D, Lata S, Prakash S, Shapiro S (2014). Discovery of new risk loci for IgA nephropathy implicates genes involved in immunity against intestinal pathogens. Nat Genet.

[3] Roberts I, Cook H, Troyanov S, Alpers C, Amore A, Barratt J, Berthoux F, Bonsib S, Bruijn J, Cattran D (2009). The Oxford classification of IgA nephropathy: pathology definitions, correlations, and reproducibility. Kidney Int.

[4] Xu X, Wang G, Chen N, Lu T, Nie S, Xu G, Zhang P, Luo Y, Wang Y, Wang X (2016). Long-term exposure to air pollution and increased risk of membranous nephropathy in China. J Am Soc Nephrol.

[5] Saeki T, Nishi S, Imai N, Ito T, Yamazaki H, Kawano M, Yamamoto M, Takahashi H, Matsui S, Nakada S (2010). Clinicopathological characteristics of patients with IgG4-related tubulointerstitial nephritis. Kidney Int.

[6] Raissian Y, Nasr S, Larsen C, Colvin R, Smyrk T, Takahashi N, Bhalodia A, Sohani A, Zhang L, Chari S (2011). Diagnosis of IgG4-related tubulointerstitial nephritis. J Am Soc Nephrol.

[7] Lin W, Lu S, Chen H, Wu Q, Fei Y, Li M, Zhang X, Tian X, Zheng W, Leng X (2015). Clinical characteristics of immunoglobulin G4-related disease: a prospective study of 118 Chinese patients. Rheumatology (Oxford).

[8] Cornell L (2012). IgG4-related kidney disease. Semin Diagn Pathol.

[9] Wang G, Chen Y, Cheng H, Xu X, Sun L, Dong H (2019). Antineutrophil cytoplasmic antibody and/or antiglomerular basement membrane antibody associated crescentic glomerulonephritis in combination with IgG4-related tubulointerstitial nephritis. Clin Exp Rheumatol.

[10] Kawano M, Saeki T (2015). IgG4-related kidney disease--an update. Curr Opin Nephrol Hypertens.

[11] Yu M, Kim Y, Koo H, Chin H (2017). Short-term anti-proteinuric effect of tacrolimus is not related to preservation of the glomerular filtration rate in IgA nephropathy: a 5-year follow-up study. PLoS One.

[12] Wallace Z, Naden R, Chari S, Choi H, Della-Torre E, Dicaire J, Hart P, Inoue D, Kawano M, Khosroshahi A (2020). The 2019 American College of Rheumatology/European league against rheumatism classification criteria for IgG4-related disease. Ann Rheum Dis.

[13] Floege J, Barbour SJ, Cattran DC, Hogan JJ, Nachman PH, Tang SCW, et al. Management and treatment of glomerular diseases (part 1): conclusions from a Kidney Disease: Improving Global Outcomes (KDIGO) Controversies Conference. Kidney Int. 2019;95(2):268-280. 10.1016/j.kint.2018.10.018 30665568

[14] Tan J, Dong L, Ye D, Tang Y, Hu T, Zhong Z, et al. The efficacy and safety of immunosuppressive therapies in the treatment of IgA nephropathy: A network meta-analysis. Sci Rep. 2020;10(1):6062. 10.1038/s41598-020-63170-w PMC7142138 32269271

[15] Hassler J (2020). IgA nephropathy: a brief review. Semin Diagn Pathol.

[16] Stone J, Zen Y, Deshpande V (2012). IgG4-related disease. N Engl J Med.

[17] Kamisawa T, Zen Y, Pillai S, Stone J (2015). IgG4-related disease. Lancet..

[18] Kawano M, Saeki T, Nakashima H, Nishi S, Yamaguchi Y, Hisano S, Yamanaka N, Inoue D, Yamamoto M, Takahashi H (2011). Proposal for diagnostic criteria for IgG4-related kidney disease. Clin Exp Nephrol.

[19] Cortazar F, Stone J (2015). IgG4-related disease and the kidney. Nat Rev Nephrol.

[20] Zhang P, Cornell L (2017). IgG4-related Tubulointerstitial nephritis. Adv Chronic Kidney Dis.

[21] Wyatt R, Julian B (2013). IgA nephropathy. N Engl J Med.

[22] Saeki T, Kawano M (2014). IgG4-related kidney disease. Kidney Int.

[23] Yunyun F, Yu C, Panpan Z, Hua C, Di W, Lidan Z, Linyi P, Li W, Qingjun W, Xuan Z (2017). Efficacy of cyclophosphamide treatment for immunoglobulin G4-related disease with addition of glucocorticoids. Sci Rep.

[24] Wang Z, Yu C, Zhou L, Chen X (2017). Effects of Tripterygium wilfordii induction therapy to IgA nephropathy patients with heavy proteinuria. Biol Pharm Bull.

[25] Zheng J, Gong X, Wu Z (2020). Immunosuppressive agents in the treatment of IgA nephropathy: a meta-analysis of clinical randomized controlled literature. Niger J Clin Pract.

[26] Zhang Y, Luo J, Hu B, Ma T (2018). Efficacy and safety of tacrolimus combined with glucocorticoid treatment for IgA nephropathy: a meta-analysis. J Int Med Res.

[27] Peng W, Tang Y, Jiang Z, Li Z, Mi X, Qin W (2016). The effect of calcineurin inhibitors in the treatment of IgA nephropathy: a systematic review and meta-analysis (PRISMA). Medicine..

[28] Song Y, Cai G, Xiao Y, Wang Y, Yuan B, Xia Y, Wang S, Chen P, Liu S, Chen X (2017). Efficacy and safety of calcineurin inhibitor treatment for IgA nephropathy: a meta-analysis. BMC Nephrol.

[29] Fan L, Liu Q, Liao Y, Li Z, Ji Y, Yang Z, Chen J, Fu J, Zhang J, Kong Y (2013). Tacrolimus is an alternative therapy option for the treatment of adult steroid-resistant nephrotic syndrome: a prospective, multicenter clinical trial. Int Urol Nephrol.

[30] Takanashi S, Kaneko Y, Takeuchi T (2019). Effectiveness of tacrolimus on IgG4-related disease. Mod Rheumatol.

[31] Akiyama M, Suzuki K, Yasuoka H, Kaneko Y, Yamaoka K, Takeuchi T (2018). Follicular helper T cells in the pathogenesis of IgG4-related disease. Rheumatology (Oxford).

[32] Akiyama M, Suzuki K, Yamaoka K, Yasuoka H, Takeshita M, Kaneko Y, Kondo H, Kassai Y, Miyazaki T, Morita R (2015). Number of circulating follicular helper 2 T cells correlates with IgG4 and Interleukin-4 levels and Plasmablast numbers in IgG4-related disease. Arthritis Rheumatol.

[33] Akiyama M, Yasuoka H, Yamaoka K, Suzuki K, Kaneko Y, Kondo H, Kassai Y, Koga K, Miyazaki T, Morita R (2016). Enhanced IgG4 production by follicular helper 2 T cells and the involvement of follicular helper 1 T cells in the pathogenesis of IgG4-related disease. Arthritis Res Ther.

[34] Fernández-Codina A, Pinilla B, Pinal-Fernández I, López C, Fraile-Rodríguez G, Fonseca-Aizpuru E, Carballo I, Brito-Zerón P, Feijóo-Massó C, López-Dupla M (2018). Treatment and outcomes in patients with IgG4-related disease using the IgG4 responder index. Joint Bone Spine.

[35] Carruthers M, Stone J, Deshpande V, Khosroshahi A (2012). Development of an IgG4-RD responder index. Int J Rheumatol.

[36] Wallace Z, Khosroshahi A, Carruthers M, Perugino C, Choi H, Campochiaro C, Culver E, Cortazar F, Della-Torre E, Ebbo M (2018). An international multispecialty validation study of the IgG4-related disease responder index. Arthritis Care Res (Hoboken).

[37] Yi J, He Z, Xu S, Feng S. Efficacy and safety of leflunomide in IgA nephropathy: a systematic review and meta-analysis. Int Urol Nephrol. 2019;51(11):1987-1998. 10.1007/s11255-019-02255-6 31515666

[38] Wang Y, Li K, Gao D, Luo G, Zhao Y, Wang X, Zhang J, Jin J, Zhao Z, Yang C (2017). Combination therapy of leflunomide and glucocorticoids for the maintenance of remission in patients with IgG4-related disease: a retrospective study and literature review. Intern Med J.

